# Metabolic sinkholes: Histones as methyl repositories

**DOI:** 10.1371/journal.pbio.3002371

**Published:** 2023-10-27

**Authors:** Ansar Karimian, Maria Vogelauer, Siavash K. Kurdistani

**Affiliations:** Department of Biological Chemistry, David Geffen School of Medicine, University of California, Los Angeles, California, United States of America

## Abstract

Perez and Sarkies uncover histones as methyl group repositories in normal and cancer human cells, shedding light on an intriguing function of histone methylation in optimizing the cellular methylation potential independently of gene regulation.

S-adenosylmethionine (SAM) functions as the major methyl donor for numerous cellular reactions and processes including DNA, RNA and protein methylation, amino acid metabolism, nucleotide synthesis, neurotransmitter synthesis, and more. SAM’s role is facilitated by its readily available methyl group (CH_3_) for transfer in various methylation reactions catalysed by methyltransferase enzymes [1].

The utilization of SAM by methyltransferases generates the by-product S-adenosylhomocysteine (SAH). SAH competes with SAM for binding to methyltransferase enzymes and can inhibit the enzyme’s ability to carry out further methylation reactions. SAH itself can undergo further metabolism: it can be recycled through the methionine cycle to regenerate SAM or directed into the transsulfuration pathway, supporting the synthesis of sulfur-containing compounds such as cysteine and glutathione (Fig 1) [1]. Consequently, the SAM/SAH ratio is a critical parameter that reflects the cellular capacity for methylation reactions as well as the metabolic pathways reliant on SAH.

**Fig 1:**
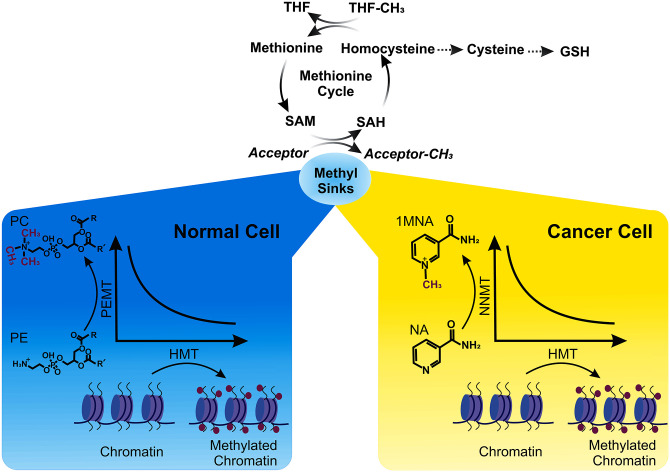
Histones as sinks for methyl groups and their interplay with other methyl sinks. In the methionine cycle, SAM conversion to SAH is contingent on the presence of methyl acceptors, often referred to as methyl sinks. SAH can be further metabolized through the transsulfuration pathway, contributing to the synthesis of essential sulfur-containing molecules such as cysteine and glutathione. Cells can methylate various substrates to serve as methyl sinks. Among these reactions, an inverse relationship exists between HMTs and NNMT, which generates 1MNA, in cancer cells, as well as between HMTs and PEMT, which generates PC, in normal cells. This suggests that histone methylation by HMTs can be employed as an alternative strategy to maintain an appropriate SAM/SAH ratio when NNMT or PEMT activities are low in cancer and normal cells, respectively. The choice of which pathway to utilize may be influenced by factors including the capacity and limitations of each methyl sink, the downstream functions of the methylated reaction products, the fate of the methyl group itself, and the subsequent metabolism of SAH. Importantly, changes in histone methylation in response to methyl sink usage do not appear to correlate with alterations in the expression of genes typically associated with histone methylation. THF–tetrahydrofolate; R and R’ = fatty acids.

Balancing the SAM/SAH ratio hinges partly on substrates capable of accepting SAM’s methyl group, known as “methylation sinks” (Fig 1). Notable reactions that involve SAM utilization and result in its diminished levels include the synthesis of 1-methylnicotinamide (1MNA) from nicotinamide (NA) via nicotinamide N-methyltransferase (NNMT), and the conversion of phosphatidylethanolamine (PE) into phosphatidylcholine (PC) by phosphatidylethanolamine methyltransferase (PEMT) [2].

Histones, boasting numerous lysine residues susceptible to methylation and an array of histone methyltransferases (HMTs), have been demonstrated as methyl sinks in budding yeast, particularly under conditions of impaired phospholipid methylation [3]. However, the question of whether histones fulfill a comparable role in other eukaryotes, including human cells, remained unanswered.

In this issue, Perez and Sarkies [4] adeptly leverage a substantial body of published data to demonstrate that histones indeed function as alternative methyl sinks to NA and PE in human cells (Fig 1). Their first key insight came from correlating the expression of HMTs with metabolite levels in cancer datasets. This analysis unveiled 1MNA as the most *negatively* correlated metabolite with the expression of most HMTs, with a similar inverse correlation observed between HMTs and NNMT, the enzyme that generates 1MNA.

These reciprocal relationships became stronger when aggregating the expression of all HMTs, suggesting that each HMT contributes to the overarching inverse relationship irrespective of its known association with gene expression or repression. In line with this, the expression of HMTs showed a positive correlation among themselves, suggesting potential coordination in their overall expression that may be linked to the activities of other methyl sink pathways.

In datasets from normal tissues, the expression of HMTs exhibited a weaker negative correlation with NNMT but a stronger one with PEMT. Once again, the aggregate expression of HMTs displayed the strongest inverse relationship with PEMT, suggesting the coordinate activity of HMTs as the pivotal factor. Nonetheless, there was some overlap observed. Some cancers displayed negative correlations between HMTs and PEMTs, while certain normal tissues exhibited negative correlations between HMTs and NNMTs.

These findings suggest that different cells and tissues, perhaps depending on metabolic or physiologic contexts, may rely on NNMT or PEMT to reduce SAM levels and on histone methylation activity as an alternative strategy when NNMT or PEMT expression is low (Fig 1). Consistent with this, the authors observed that genome-wide histone methylation levels on most lysine residues in histones H3 or H4 are also inversely related to NNMT or PEMT expression, albeit with some exceptions that may be attributed to other roles of histone methylation in specific cell types.

Remarkably, the presence of either high levels of histone methylation or NNMT/PEMT (indicating low levels of histone methylation) did not correlate with changes in the transcription of genes associated with histone methylation. This suggests that the overall level of histone methylation is more indicative of the equilibrium between the activities of different methyl sink pathways rather than gene expression.

After noting the surprising coordinated expression of HMTs in the human datasets, the authors turned to data from the worm *Caenorhabditis elegans* to explore potential mechanisms governing the co-regulation of HMTs. They discovered intriguing evidence suggesting that HMTs might be subject to coordinated regulation by the Retinoblastoma protein (Rb) and E2F transcription factors, well-known regulators of the cell cycle [5]. Consistently, the promoters of human HMT genes were found to be enriched with E2F1 transcription factor binding sites. This potential regulation of HMTs by the Rb/E2F axis also establishes a link between SAM/SAH ratio, HMT activities, and cell cycle progression.

Furthermore, this analysis allowed the authors to propose a model for how the inverse relationship between HMTs and NNMT may be achieved. Their analyses suggested that the Rb/E2F activity maintains the suppression of HMTs, which, in turn, likely suppress NNMT expression. Consequently, when HMT activities, and thus histone methylation, are engaged, the NNMT pathway is down-regulated, thereby ensuring reciprocal activities of these 2 methyl sink pathways. Although the regulation of HMTs may differ across various organisms, histones functioning as a metabolic sink for methyl groups may be a common phenomenon in eukaryotes.

With the presence of various methyl group sinks, an important question arises: What factors or conditions determine the preferential utilization of one sink pathway over another? While the answer is not known, it may lie in the capacity and limitations of each methyl sink, the downstream functions of the methylated reaction products including the methyl group itself as well as the subsequent metabolism of SAH.

For instance, it is conceivable that the production of SAH as a particular consequence of histone methylation may fuel syntheses of cysteine and glutathione, which are critical for highly proliferative cells such as cancer cells [6]. Supporting this notion is the positive correlation of HMT expression and cystathionine, a precursor metabolite to cysteine and glutathione. Potentially related to this, a recent study demonstrated that impairing the function of the SWI/SNF chromatin remodeling complex induces a cysteine-deficient phenotype [7]. Taken together, these findings suggest that histones may play a direct role in regulating sulfur homeostasis.

Increasing evidence suggests that chromatin functions as a metabolic organelle. It not only receives signals from the cell, in the form of small molecules that modify histones for example, but also provides instructive signals back to govern cellular metabolism and physiology. Chromatin may serve as a reservoir of acetate [8,9], a repository of methyl groups [3,4], or source of cuprous (Cu^1+^) ions, the latter facilitated by the copper reductase activity of histone H3 [10]. These functions seemingly operate independently of conventional transcriptional regulation. In fact, the observed changes in gene activity may represent adaptive responses aimed at implementing and managing the metabolic functions of chromatin. This metabolic perspective on histones and chromatin prompts a reevaluation of their roles in biology and disease.
